# Supplementary material for: The Zinc-BED Transcription Factor Bedwarfed Promotes Proportional Dendritic Growth and Branching through Transcriptional and Translational Regulation in Drosophila
Source: Int J Mol Sci. 2023 Mar 28;24(7):6344. doi: 10.3390/ijms24076344 (PMC10094446; doi:10.3390/ijms24076344)
Supplement: Supplementary file 1 [file ijms-24-06344-s001.zip › Bhattacharjee_Supplementary_Figures_2023.pdf]

Supplementary Figures

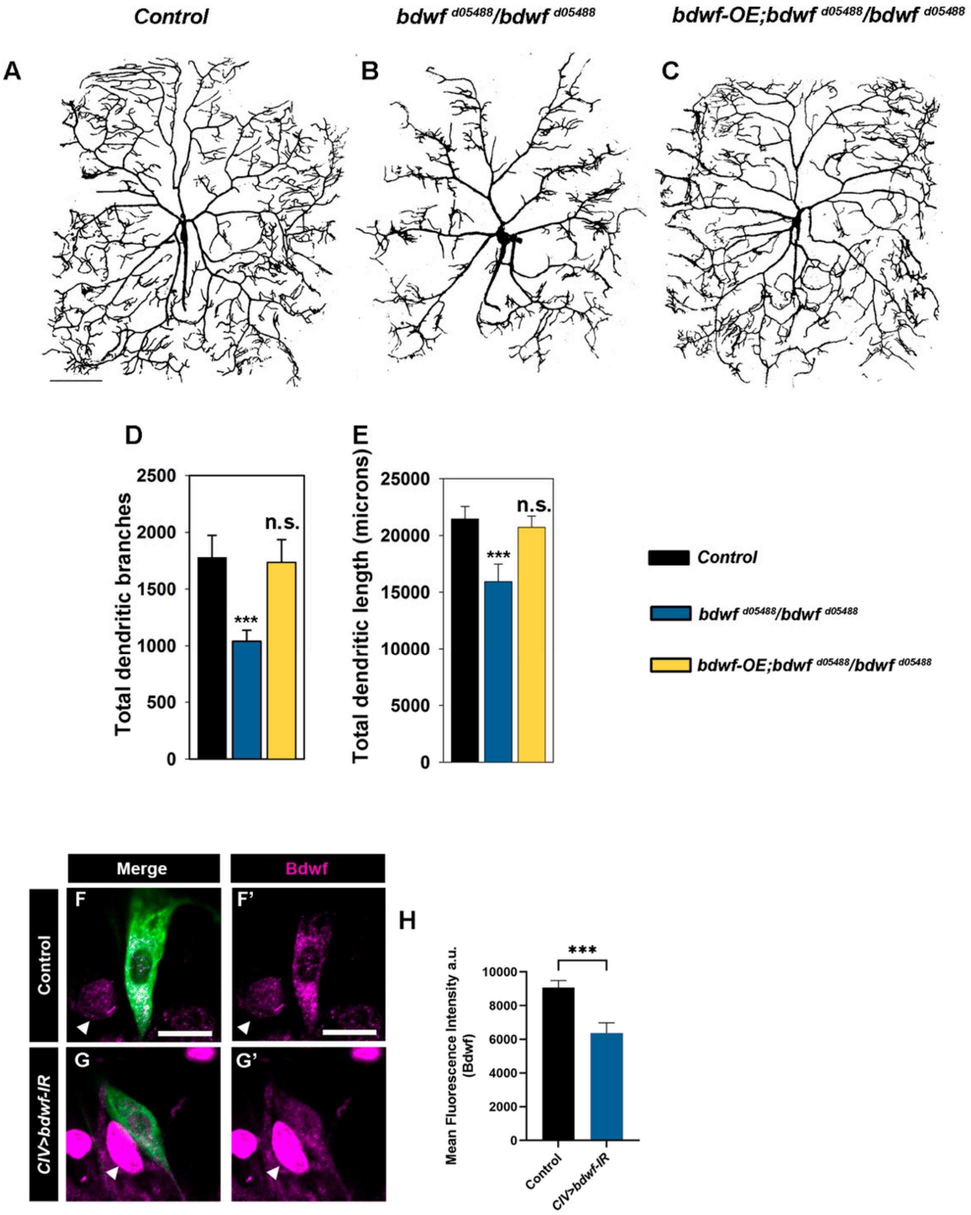

Figure S1: Rescue of bdwf mutant dendritic defects and validation of bdwf-IR and bdwf antibody. (A-E) Compared to wild-type control CIV neurons (A), *bdwf*^{d0588} homozygous mutant CIV neurons results display a strong and penetrant dendritic hypotrophy (B), which is fully rescued by CIV expression of *UAS-bdwf* (C, D, E). White arrowheads point to Bdwf expression in non-neuronal cell types (*e.g.* epithelia or muscle) Genotypes: (A- E) *Gal4*^{477}*, UAS-mCD8::GFP/+; +/+* or *bdwf*^{d0588}*/ bdwf*^{d0588} or *UAS-Bdwf-FLAG-HA/ +.* Scale bar, 50 μm, n=8 neurons per genotype. (F-H) *bdwf-IR* knockdown in CIV neurons leads to a significant

reduction in Bdwf protein expression levels. Scale bar, 10 µm. n= 16 (control) and n=9 (*bdwf-IR)*. Error bars represent +/- S.E.M. ***p≤0.01 (Student's t-test).

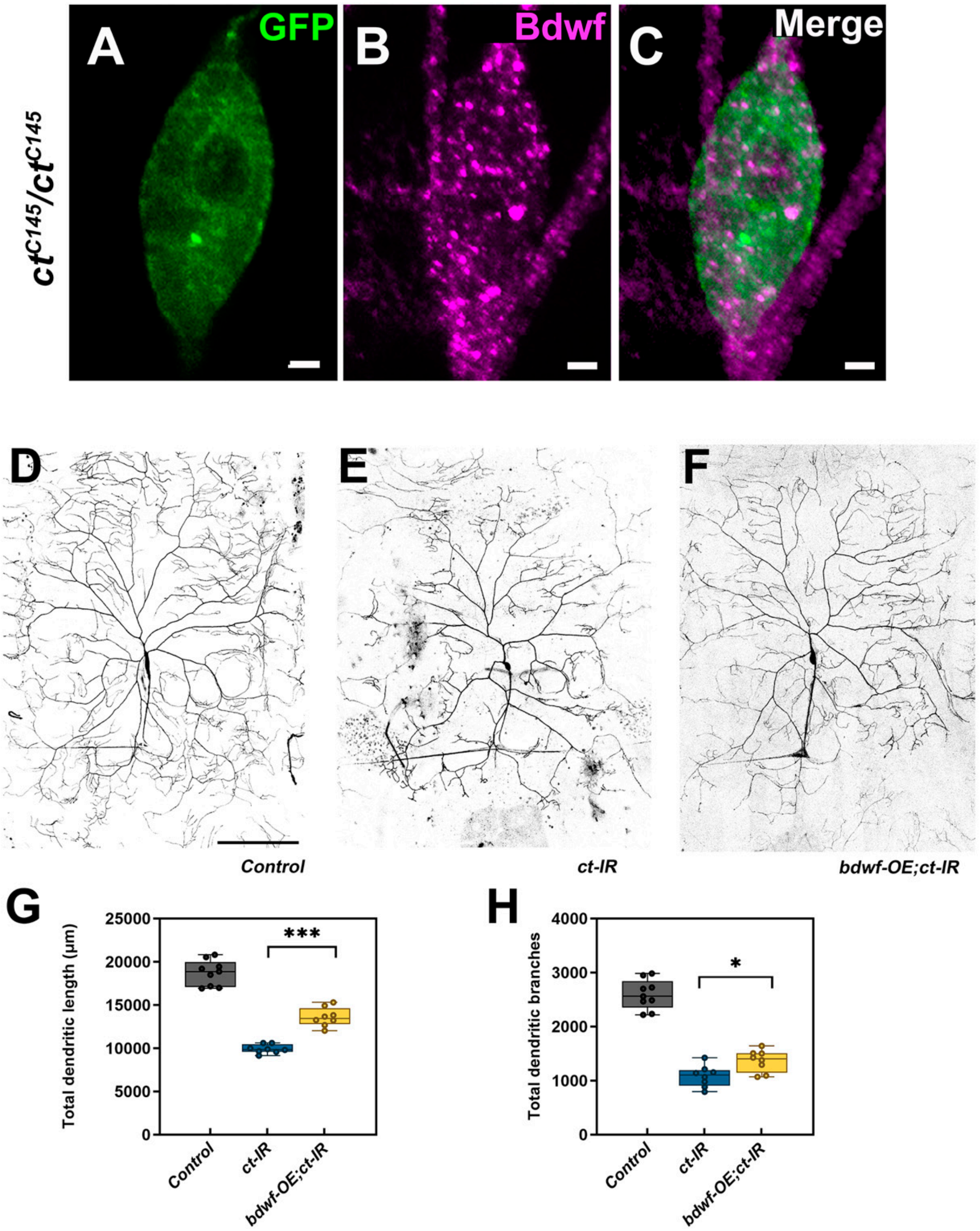

Figure S2: Cut is not required for Bdwf expression and Bdwf can partially rescue cut RNAi-mediated defects in CIV dendrite morphogenesis. (A-C) Representative images of *ct* MARCM clones of CIV md neurons stained if CD8 (green) and Bdwf (magenta) showing normal Bdwf expression in *ct* mutants. Scale bar: 1 µm. (D-F) Bdwf acts downstream of Ct to promote dendritic growth in CIV md neurons. Compared to control (D), *ct-IR* (E, G, H) shows significant reduction in both the total dendritic length and the total dendritic branches which is partially rescued by the simultaneous co-expression of *UAS- bdwf-FLAG-HA* and *ct-IR* (F, G, H) in CIV ddaC neurons. Scale bar, 100 µm. (G, H) Quantification of TDL and TDB for CIV da neurons. Error

bars represent +/- SEM, * $p \leq 0.05$, *** $p \leq 0.001$ (One-way ANOVA with Sidak's multiple comparison test). n=9 for WT, n=8 for *ct-IR*, n=8 for *bdwf-OE+ct-IR*. Genotypes: *$GAL4^{477}$,UAS-mCD8::GFP/+;UAS-CD4-tdTOM/+* (D, G, H), *$GAL4^{477}$,UAS-mCD8::GFP/+;UAS-ct-IR/ UAS-CD4-tdTOM* (E, G, H), *$GAL4^{477}$,UAS-mCD8::GFP/+;UAS-ct-IR/ UAS- bdwf-FLAG-HA* (F, G, H).
